# Costal chondrosarcoma in a woman with hereditary multiple exostoses - a case report

**DOI:** 10.3389/fonc.2025.1469072

**Published:** 2025-02-17

**Authors:** Ze Yang, Kaiqiang Wang, Jiangtao Pu

**Affiliations:** Department of Thoracic Surgery, Affiliated Hospital of Southwest Medical University, Luzhou, China

**Keywords:** hereditary multiple exostoses, malignant conversion, costal chondrosarcoma, mediastinal tumor, treatment

## Abstract

In this report, we present a case of a 32-year-old female previously diagnosed with hereditary multiple exostoses(HME) who was incidentally found to have an asymptomatic anterior mediastinal mass during a routine examination. Computed tomography imaging revealed a well-defined mass measuring approximately 2.3 cm x 4.0 cm x 4.7 cm in the anterior mediastinum with multiple nodular areas of high density within. The mass caused compression and narrowing of the right ventricle. The patient subsequently underwent intralesional resection of the tumor, and histopathological examination confirmed a diagnosis of well-differentiated chondrosarcoma. Given the patient’s medical history, the chondrosarcoma was suspected to have originated from malignant transformation of a rib osteochondroma. The patient received adjuvant radiotherapy postoperatively and has been followed up for one year with no evidence of recurrence. This case reports a highly rare costal chondrosarcoma secondary to hereditary multiple exostoses, located in the anterior mediastinum and compressing the right ventricle. To our knowledge, this is the first reported case of costal chondrosarcoma secondary to HME occurring in the anterior mediastinum, which requires differentiation from other common anterior mediastinal tumors.

## Introduction

Chondrosarcoma is a relatively common malignant bone tumor originating from cartilage, accounting for approximately 20-30% of all primary malignant bone tumors in the United States, with an annual incidence of 1 to 4 cases per million population, depending on geographic and population differences (1, 2). A subset of chondrosarcomas arises from pre-existing benign conditions, such as hereditary multiple exostoses (HME), and is referred to as secondary chondrosarcoma. HME, an autosomal dominant genetic disorder, is characterized by the formation of multiple osteochondromas throughout the skeleton. These osteochondromas predominantly affect the metaphyses of long bones, the scapulae, and the pelvis, while rib involvement is comparatively rare (3). The cause of HME is linked to mutations in the EXT1 and EXT2 genes. These mutations disrupt the synthesis and elongation of heparan sulfate (HS), a glycosaminoglycan essential for regulating growth factor signaling pathways such as Indian hedgehog (IHH), fibroblast growth factors (FGFs), and bone morphogenetic proteins (BMPs). The impaired HS biosynthesis affects chondrocyte differentiation and proliferation, leading to the formation of osteochondromas (4–6). The most serious complication of HME is malignant transformation into chondrosarcoma, with previous studies estimating the incidence of malignant transformation to be approximately 1-5% (7, 8). This transformation is more commonly observed in osteochondromas located in the pelvis and proximal femur, and less frequently in the ribs (9). Costal chondrosarcoma caused by HME is very rare, with only a few reports available. For example, Acharya et al. reported a large chondrosarcoma in the anterior chest wall from a rib osteochondroma, and Liu et al. described a similar case in the posterior chest wall (10, 11). To our knowledge, there are no prior reports of costal chondrosarcoma secondary to HME occurring in the anterior mediastinum, which presents significant diagnostic challenges due to its rarity and the need to differentiate it from more common mediastinal tumors. In this study, we present an extremely rare case of costal chondrosarcoma located in the anterior mediastinum secondary to HME.

## Case presentation

**Figure 1:**
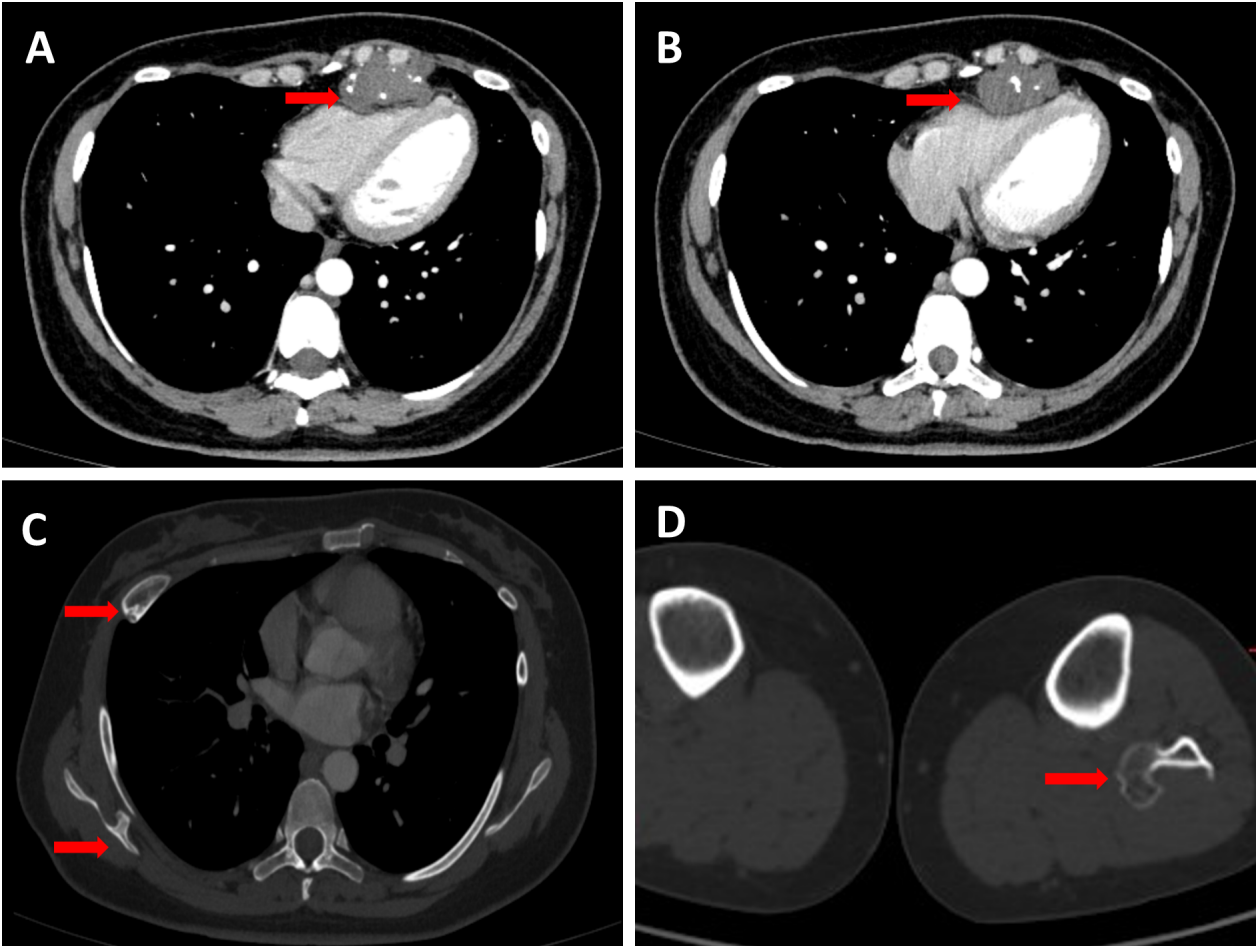
**(A,B)** Red arrows indicate the tumor in the anterior mediastinum with visible calcifications and clear edges. The tumor is pressing on the right ventricle, making it smaller. **(C)** Two red arrows highlight significant findings: one indicates a focal bony expansion on the right fifth anterior rib, suggesting a potential osteochondroma; the other points to a typical osteochondroma located on the right scapula. **(D)** The red arrow highlights an osteochondroma situated at the proximal end of the left fibula.

The case involves a 32-year-old female patient who presented to our hospital in July 2023 after a routine health checkup revealed an anterior mediastinal mass. Despite the presence of the mass, she did not exhibit any symptoms typically associated with such findings, such as chest pain, cough, fever, or shortness of breath. A contrast-enhanced computed tomography (CT) scan of her chest demonstrated a well-defined mass measuring approximately 2.3 cm x 4.0 cm x 4.7 cm in the anterior mediastinum, causing compression of the right ventricle. The mass had multiple nodular high-density areas within it (**Figures 1A, B**). Bone abnormalities were observed, including a bony prominence on the right scapula, a bony prominence on the proximal left fibula, and localized bone expansion of the right fifth anterior rib, which were all considered to be potential osteochondromas (**Figures 1C, D**). Cardiac assessments, including electrocardiogram and echocardiogram, showed T-wave changes and mild tricuspid valve regurgitation, respectively. A whole-body bone scan ruled out distant bone metastasis, and laboratory tests including complete blood count, alkaline phosphatase (ALP), and lactate dehydrogenase (LDH) levels were unremarkable. Physical examination and abdominal ultrasound did not reveal any significant abnormalities.

**Figure 2:**
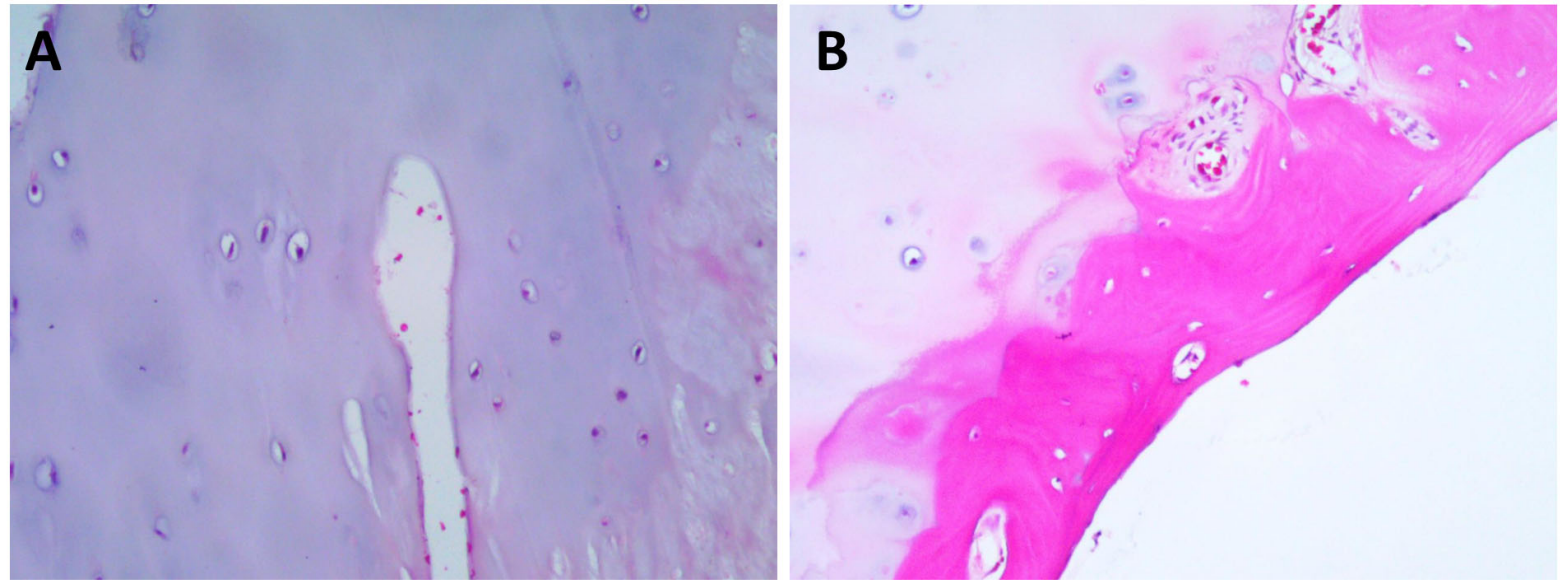
Hematoxylin and Eosin (HEx200) staining results. **(A)** HE Staining reveals the characteristic cartilage cap. **(B)** HE Staining delineates the cartilaginous cap and the adjacent fibrous layer, highlighting the distinct histological attributes of the tumor.

The patient had a significant surgical history. Four years prior, she presented to our orthopedic department with bilateral knee pain. X-rays of the knee joints revealed bone protrusions at the distal femur, right proximal tibia, consistent with osteochondromas. The lesions caused significant knee pain, which led to the decision to surgically excise them. Histopathological examination of the excised tissue confirmed the diagnosis of osteochondroma (**Figures 2A, B**). The patient’s father had hereditary multiple exostoses, suggesting a genetic predisposition. The diagnosis of HME is typically based on clinical, radiological, and histological findings. Although genetic testing was not performed at that time, the patient was diagnosed with HME given her family history, imaging results, and postoperative pathological findings, which provided sufficient evidence for the diagnosis (4, 7).

**Figure 3:**
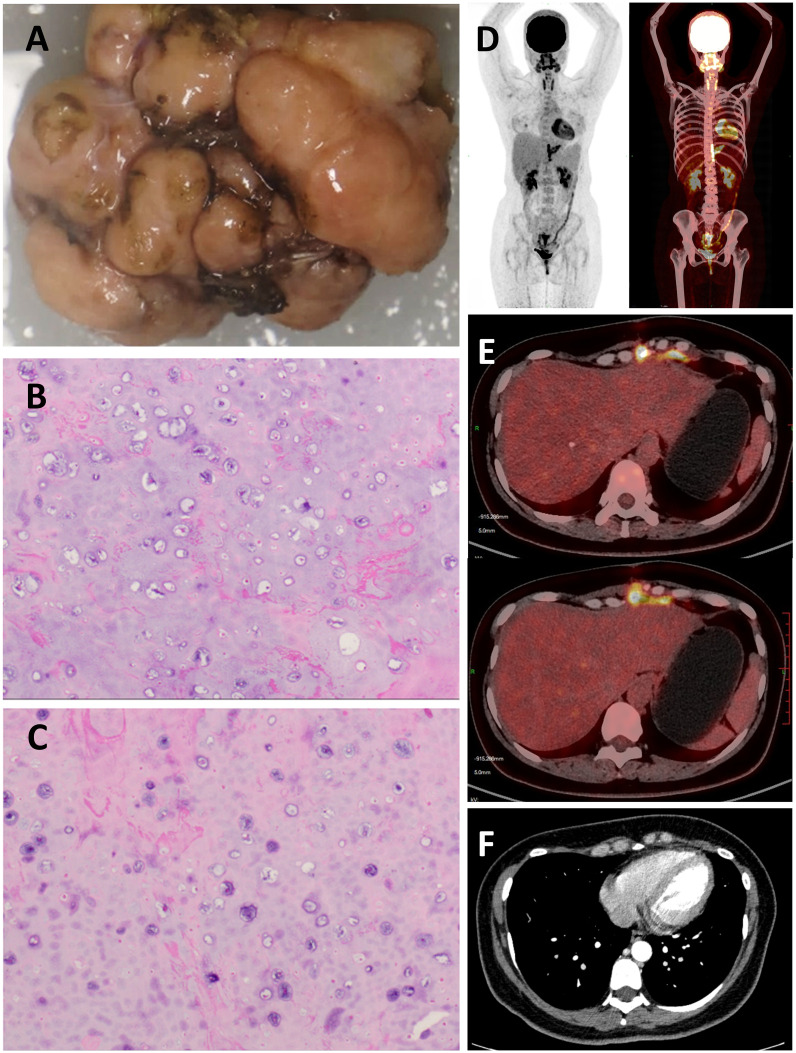
**(A)** Macroscopic Examination: The excised tumor, exhibiting a multinodular surface and a hard consistency, measured 2.3 cm × 4.0 cm × 4.7cm. **(B,C)** Histopathological Examination (Hematoxylin and Eosin Stain): Micrographs at high magnification revealed chondrocyte hyperplasia within the cartilaginous matrix, indicative of a malignant process. **(D)** No distant metastatic lesions were identified upon PET-CT examination. **(E)** Increased glucose metabolism was observed in the surgical site on PET-CT imaging, consistent with postoperative changes. **(F)** Contrast-enhanced chest CT during postoperative follow-up.

To remove the tumor and relieve its compression on the heart, the patient underwent resection of the mediastinal tumor after obtaining informed consent: a left subxiphoid incision was made, with a 4 cm incision along the rib arch, assisted by thoracoscopy. The tumor was excised without extensive resection, preserving adjacent ribs and sternum. Intraoperatively, a hard, popcorn-like mass was observed in the left anterior mediastinum, with unclear demarcation from the rib cartilage and anterior chest wall but relatively clear relation to the pericardium and surrounding structures, suggesting that the tumor likely originated from the rib rather than from the mediastinum itself (**Figure 3A**). Postoperative histopathological examination suggested a diagnosis of well-differentiated chondrosarcoma, this was consistent with the tumor features we observed during surgery. (**Figures 3B, C**). The patient recovered and was discharged on the third postoperative day. One month after the surgery, the patient consulted the oncology department at our hospital for a follow-up and to determine the next steps for treatment. A PET-CT scan was performed to assess for potential distant metastases, and fortunately, it revealed no evidence of distant metastasis (**Figure 3D**). There was visible soft tissue swelling and increased glucose metabolism in the surgical area, with a standardized uptake value (SUV) of 8.8, indicative of postoperative changes (**Figure 3E**). Follow-up echocardiography and electrocardiogram showed no evidence of tricuspid valve regurgitation or T-wave changes, possibly due to relief of right ventricular compression post-surgery (12). CT scans confirmed that the tumor had been successfully removed, with visible improvement in right ventricular compression (**Figure 3F**). According to the NCCN guidelines, for low-grade chondrosarcomas, either wide resection or intralesional resection followed by adjuvant radiotherapy is recommended (13). Therefore, the patient received adjuvant intensity-modulated radiotherapy at our hospital. Currently, the patient has been regularly followed up for one year without evidence of tumor recurrence.

## Discussion

Chondrosarcoma is a relatively common malignant bone tumor originating from cartilage and is the second most common primary malignant bone tumor after osteosarcoma (2). Chondrosarcomas can be classified into conventional chondrosarcoma and special subtypes, with the conventional type comprising approximately 85% of all cases. Conventional chondrosarcoma can be further divided into primary chondrosarcoma and secondary chondrosarcoma, which arises from benign conditions such as hereditary multiple exostoses, Ollier disease (multiple enchondromatosis), and Maffucci syndrome.

Hereditary multiple exostoses (HME), also known as hereditary multiple osteochondromas (HMO), is a rare autosomal dominant genetic disorder with an incidence of approximately 1 in 50,000 among Caucasians (14). Current research indicates that mutations in the EXT1 and EXT2 tumor suppressor genes from the EXT gene family are responsible for both sporadic and syndromic osteochondromas, including hereditary multiple exostoses (HME) (5, 15–17). The condition is characterized by multiple benign bone outgrowths covered with cartilage caps, primarily affecting the metaphyses of long bones in the limbs, as well as the scapula and pelvis, with relatively rare involvement of the ribs.

HME can lead to various complications, with the most serious being malignant transformation into chondrosarcoma. Previous studies have estimated the malignant transformation rate of HME to be approximately 1-5%. However, the timing of symptom development can vary depending on the tumor’s location. Early signs of malignant transformation of HME can be detected through imaging techniques such as CT and MRI (18, 19). Fortunately, secondary chondrosarcomas are generally low-grade and have a lower rate of metastasis, which aligns with the condition of the patient we report here (20, 21). The risk of malignant transformation is related to the location of the osteochondromas; those located in the pelvis and proximal femur are more prone to malignant transformation, while malignant transformation of rib osteochondromas into chondrosarcoma is exceedingly rare, with only a few cases reported in the literature. Malignant transformation is often initially detected by clinical symptoms, such as growth and pain occurring after puberty. However, the timing of symptom development can vary depending on the tumor’s location. Metesh Acharya et al. reported a case of a large chondrosarcoma primarily located in the anterior chest wall originating from a rib (10). Similarly, Wenliang Liu et al. reported a case of a large chondrosarcoma primarily located in the posterior chest wall, both believed to have arisen from malignant transformation of HME (11). In contrast, our patient benefited from early detection, with a relatively small tumor size. However, the growth pattern of the tumor in this case extended into the thoracic cavity, directly compressing the heart and significantly narrowing the right ventricle. Additionally, due to the tumor’s location in the anterior mediastinum, the diagnosis was challenging as it needed to be differentiated from other common anterior mediastinal tumors. These include teratomas, lymphomas, and other cartilage-origin tumors such as chondroblastomas (22).

The treatment strategy for chondrosarcoma depends on the tumor’s histological grade and location. Well-differentiated (low-grade) chondrosarcomas typically grow slowly and have a relatively low recurrence rate. Due to the rarity of costal chondrosarcoma, there is no standardized treatment protocol for this condition. According to the NCCN guidelines, the preferred approach for low-grade chondrosarcomas is either wide resection or intralesional resection followed by adjuvant therapy to minimize recurrence risk. However, wide resection often requires extensive removal of adjacent chest wall structures, such as the sternum and rib cartilage, which necessitates complex reconstruction. In this case, intralesional resection was chosen to preserve the structural integrity of the chest wall, as wide resection would have caused substantial disruption to surrounding structures. Recognizing the increased risk of local recurrence with intralesional resection, the patient underwent adjuvant intensity-modulated radiotherapy (IMRT). While chondrosarcomas are traditionally considered radioresistant, recent advancements in radiotherapy, including IMRT, have shown improved potential for local control (23, 24).

In summary, we report a highly rare case of costal chondrosarcoma secondary to HME, located in the anterior mediastinum and causing compression of the right ventricle, which required differentiation from other common anterior mediastinal tumors. This case underscores the importance of routine imaging follow-up for patients with HME to enable early detection of malignant transformation. It also highlights the critical role of a multidisciplinary team, including surgeons, oncologists, radiologists, and pathologists, in improving diagnostic accuracy, surgical planning, and postoperative management (3, 25). To our knowledge, this is the first reported case of costal chondrosarcoma secondary to HME occurring in the anterior mediastinum. Our experience highlights the need to suspect secondary chondrosarcoma in HME patients when a mediastinal mass with calcified foci is detected, especially if the mass is in close proximity to the chest wall.

## Data Availability

The raw data supporting the conclusions of this article will be made available by the authors, without undue reservation.
